# Supplementary material for: The interactome of intact mitochondria by cross-linking mass spectrometry provides evidence for coexisting respiratory supercomplexes
Source: Mol Cell Proteomics. 2017 Dec 8;17(2):216–32. doi: 10.1074/mcp.RA117.000470 (PMC5795388; doi:10.1074/mcp.RA117.000470)
Supplement: Supplemental Data [file supp_RA117.000470_133922_0_supp_23978_fzffwf.zip › spectra_annotation/mito_spectra_annotation/350-1-2-1-1-1.pdf]

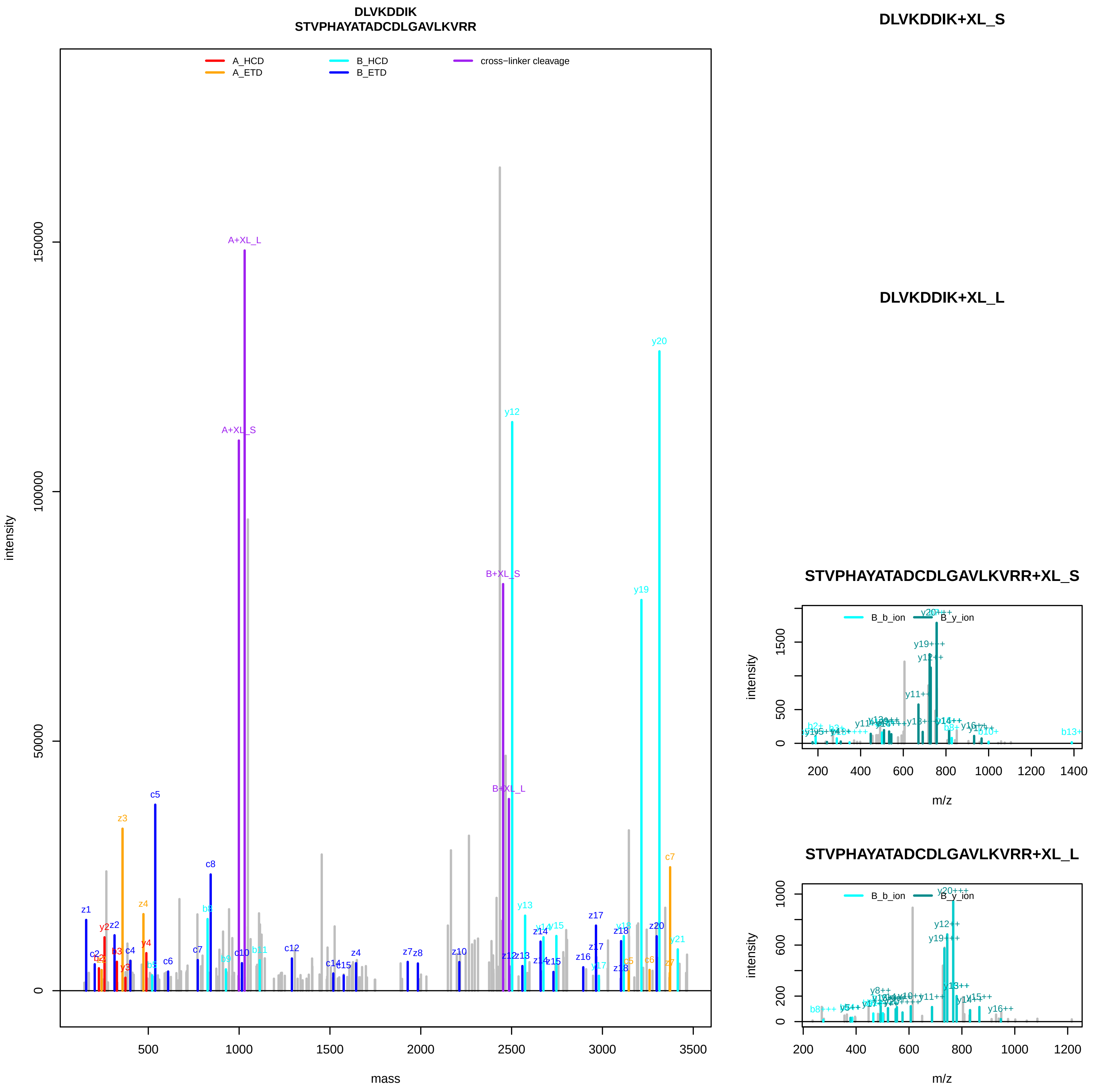

DLVKDDIK
STVPHAYATADCDLGAVLKVRR
A_HCD
A_ETD
B_HCD
B_ETD
cross−linker cleavage
A+XL_L
A+XL_S
B+XL_S
B+XL_L
y20
y12
y19
c5
z3
c8
c7
z1
z2
c2
y2
b2
b3
y3
c4
z4
y4
c6
b8
b9
c10
b11
c12
c14
c15
z7
z8
z10
y13
z12
z13
z14
y14
y15
z15
z16
z17
y17
y18
z18
c5
c6
z20
y21
c7
z7
intensity
mass
0
50000
100000
150000
500
1000
1500
2000
2500
3000
3500
DLVKDDIK+XL_S
DLVKDDIK+XL_L
STVPHAYATADCDLGAVLKVRR+XL_S
B_b_ion
B_y_ion
y20+++
y19+++
y12++
y11++
b13+
intensity
m/z
0
500
1500
200
400
600
800
1000
1200
1400
STVPHAYATADCDLGAVLKVRR+XL_L
B_b_ion
B_y_ion
y20+++
y12++
y19+++
y13++
y15++
y16++
intensity
m/z
0
200
600
1000
200
400
600
800
1000
1200
